# 4-Chloro-2-((*E*)-{3-[1-(hydroxy­imino)eth­yl]phen­yl}imino­meth­yl)phenol

**DOI:** 10.1107/S1600536809045942

**Published:** 2009-11-07

**Authors:** Li Xu, Lei Wu

**Affiliations:** a School of Chemical and Biological Engineering, Lanzhou Jiaotong University, Lanzhou 730070, People’s Republic of China

## Abstract

The title compound, C_15_H_13_ClN_2_O_2_, adopts an *E* conformation with respect to the azomethine C=N bond. The aniline and phenol rings are almost coplanar, making a dihedral angle of 3.33 (2)°. In the crystal, the mol­ecules lie about inversion centers, forming dimers that are connected by inter­molecular O—H⋯N hydrogen bonds, resulting in six-membered rings with graph-set motif *R*_2_^2^(6). In addition, there is a strong inter­molecular O—H⋯N hydrogen-bonding inter­action, resulting in an *S*(6) ring motif. Weak π–π inter­actions between the benzene rings [centroid–centroid distance = 3.809 (1) Å] further stabilize the crystal structure.

## Related literature

For background to Schiff bases, see: Dong *et al.* (2007 ▶, 2008 ▶, 2009 ▶); Eltayeb *et al.* (2008 ▶). For related crystal strcutures, see: Butcher *et al.* (2005 ▶); Golovnia *et al.* (2009 ▶); Xu *et al.* (2008 ▶); Rafiq *et al.* (2008 ▶); Zhao *et al.* (2009 ▶).
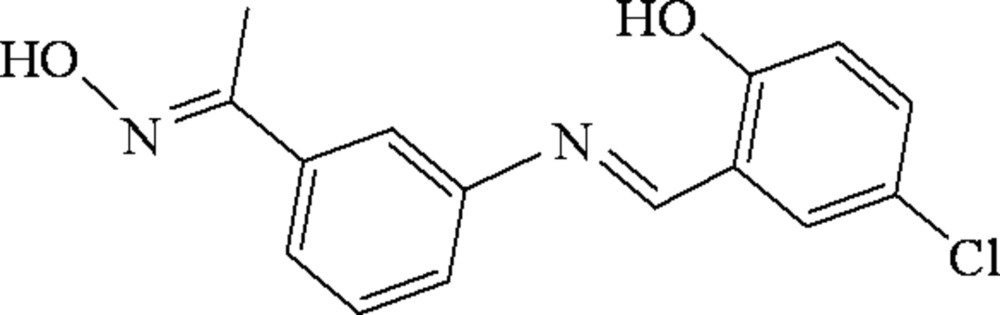


## Experimental

### 

#### Crystal data

C_15_H_13_ClN_2_O_2_
*M* *_r_* = 288.72
Monoclinic, 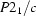

*a* = 16.7139 (16) Å
*b* = 5.9983 (6) Å
*c* = 13.3902 (11) Å
β = 96.328 (2)°
*V* = 1334.3 (2) Å^3^
*Z* = 4
Mo *K*α radiation
μ = 0.29 mm^−1^
*T* = 298 K
0.40 × 0.12 × 0.07 mm

#### Data collection

Siemens SMART 1000 CCD area-detector diffractometer
Absorption correction: multi-scan (*SADABS*; Sheldrick, 1996 ▶) *T*_min_ = 0.893, *T*_max_ = 0.980
6410 measured reflections
2349 independent reflections
1398 reflections with *I* > 2σ(*I*)
*R*_int_ = 0.053

#### Refinement

*R*[*F* ^2^ > 2σ(*F* ^2^)] = 0.044
*wR*(*F* ^2^) = 0.092
*S* = 1.04
2349 reflections
182 parameters
H-atom parameters constrained
Δρ_max_ = 0.17 e Å^−3^
Δρ_min_ = −0.18 e Å^−3^

### 

Data collection: *SMART* (Siemens, 1996 ▶); cell refinement: *SAINT* (Siemens, 1996 ▶); data reduction: *SAINT*; program(s) used to solve structure: *SHELXS97* (Sheldrick, 2008 ▶); program(s) used to refine structure: *SHELXL97* (Sheldrick, 2008 ▶); molecular graphics: *SHELXTL* (Sheldrick, 2008 ▶); software used to prepare material for publication: *SHELXTL*.

## Supplementary Material

Crystal structure: contains datablocks global, I. DOI: 10.1107/S1600536809045942/pv2220sup1.cif

Structure factors: contains datablocks I. DOI: 10.1107/S1600536809045942/pv2220Isup2.hkl

Additional supplementary materials:  crystallographic information; 3D view; checkCIF report

## Figures and Tables

**Table 1:** Hydrogen-bond geometry (Å, °)

*D*—H⋯*A*	*D*—H	H⋯*A*	*D*⋯*A*	*D*—H⋯*A*
O2—H2⋯N2	0.82	1.87	2.601 (3)	147
O1—H1⋯N1^i^	0.82	2.06	2.789 (3)	149

Symmetry code: (i) 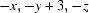.

**Fig. 1.**
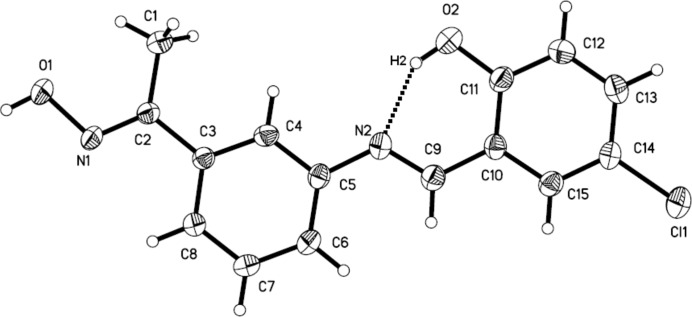
The molecule structure of the title compound with atomic numbering scheme. Displacement ellipsoids are drawn at the 30% probability level.

**Fig. 2.**
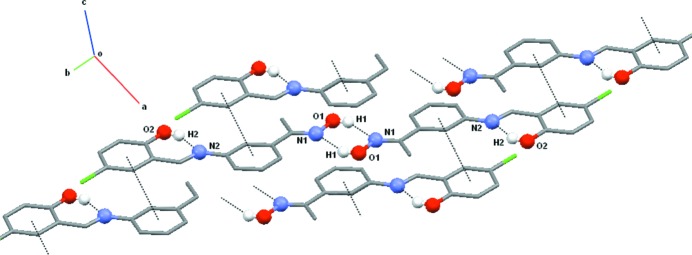
Part of the supramolecular structure of the title compound, showing a dimer formed by intermolecular O—H···O and O—H···N hydrogen bonds as well as π–π stacking interactions. H atoms not involved in hydrogen bonding have been omitted for clarity.

## supplementary crystallographic information

### Comment

Schiff base ligands have numerous applications in chemistry, biology, physics and advanced materials and catalysis (Dong *et al.*, 2007; Dong *et al.*, 2008; Eltayeb *et al.*, 2008). The pressence of Schiff base functional group together with oxime (–C═N—OH) may result in significant increase of chelating efficiency and ability to form polynuclear complexes (Golovnia *et al.*, 2009; Dong *et al.*, 2009; Xu *et al.*, 2008). Owing to the importance of oxime-type compounds, we report in this article the synthesis and crystal structure of the title compound, (I), which contains both the functional groups.

In the structure of the title compound (Fig. 1), the bond lengths and bond angles are in normal ranges and agree well with the coresponding bond lengths and angles reported for the crystal structures related to the title compound, e.g., (Butcher *et al.*, 2005; Golovnia *et al.*, 2009; Xu *et al.*, 2008; Rafiq *et al.*, 2008; Zhao *et al.*, 2009). The molecule of (I) adopts an E conformation with respect to the azomethine C═N bond. The aniline (C3—C8) and phenol rings (C10—C15) are almost coplanar with each other, making a dihedral angle of 3.33 (2)°; the torsion angles O1—N1—C2—C3 and C5—N2—C9—C10 are 178.4 (2) and -178.9 (2)°, respectively. The molecules of (I) lie about inversion centers forming dimers that are connected by intermolecular hydrogen bonds of the type O—H···N resulting in six-membered rings which can be described in graph-set notation as *R*_2_^2^(6) motif. In addition, there is a strong intermolecular hydrogen bonding interaction of the type O—H···N resulting in an S(6) ring motif (Table 1). Moreover, weak π–π interactions between the benzene rings (centroid-centroid distance = 3.809 (1) Å) further stabilize the crystal structure (Fig. 2).

### Experimental

To an ethanol solution (5 ml) of 3-aminophenylethanone oxime (150.2 mg, 1.00 mmol) was added dropwise an ethanol solution (5 ml) of 5-chlorinebenzaldehyde (156.8 mg, 1.00 mmol). Immediately, a yellow precipitate was obtained. The mixture solution was stirred at 328–333 K for 5 h. After cooling to room temperature, the precipitate was filtered off, dried *in vacuo* and purified by recrystallization from ethanol to a solid material. Yellow needle-like single crystals suitable for X-ray diffraction studies were obtained by slow evaporation from a solution of dichloromethane at room temperature in about two weeks.

### Refinement

H atoms were treated in a riding mode with distances C—H = 0.96 Å (CH_3_), 0.93 Å (CH) and O—H= 0.82 Å. The isotropic displacement parameters for all H atoms were set equal to 1.2 or 1.5 *U*_eq_ of the carrier atom.

### Crystal data

**Table:**

C_15_H_13_ClN_2_O_2_	*F*(000) = 600
*M**_r_* = 288.72	*D*_x_ = 1.437 Mg m^−^^3^
Monoclinic, *P*2_1_/*c*	Melting point = 454–456 K
Hall symbol: -P 2ybc	Mo *K*α radiation, λ = 0.71073 Å
*a* = 16.7139 (16) Å	Cell parameters from 1148 reflections
*b* = 5.9983 (6) Å	θ = 3.1–25.3°
*c* = 13.3902 (11) Å	µ = 0.29 mm^−^^1^
β = 96.328 (2)°	*T* = 298 K
*V* = 1334.3 (2) Å^3^	Needle, yellow
*Z* = 4	0.40 × 0.12 × 0.07 mm

### Data collection

**Table:**

Siemens SMART 1000 CCD area-detector diffractometer	2349 independent reflections
Radiation source: fine-focus sealed tube	1398 reflections with *I* > 2σ(*I*)
graphite	*R*_int_ = 0.053
φ and ω scans	θ_max_ = 25.0°, θ_min_ = 2.5°
Absorption correction: multi-scan (*SADABS*; Sheldrick, 1996)	*h* = −18→19
*T*_min_ = 0.893, *T*_max_ = 0.980	*k* = −7→7
6410 measured reflections	*l* = −15→11

### Refinement

**Table:**

Refinement on *F*^2^	Primary atom site location: structure-invariant direct methods
Least-squares matrix: full	Secondary atom site location: difference Fourier map
*R*[*F*^2^ > 2σ(*F*^2^)] = 0.044	Hydrogen site location: inferred from neighbouring sites
*wR*(*F*^2^) = 0.092	H-atom parameters constrained
*S* = 1.03	*w* = 1/[σ^2^(*F*_o_^2^) + (0.0246*P*)^2^] where *P* = (*F*_o_^2^ + 2*F*_c_^2^)/3
2349 reflections	(Δ/σ)_max_ < 0.001
182 parameters	Δρ_max_ = 0.17 e Å^−^^3^
0 restraints	Δρ_min_ = −0.18 e Å^−^^3^

### Special details

**Table:**

Experimental. m. p. 454–456 K. Anal. Calc.: C, 62.40; H, 4.54; N, 9.70. Found: C, 62.10; H, 4.59; N, 9.89.
Geometry. All e.s.d.'s (except the e.s.d. in the dihedral angle between two l.s. planes) are estimated using the full covariance matrix. The cell e.s.d.'s are taken into account individually in the estimation of e.s.d.'s in distances, angles and torsion angles; correlations between e.s.d.'s in cell parameters are only used when they are defined by crystal symmetry. An approximate (isotropic) treatment of cell e.s.d.'s is used for estimating e.s.d.'s involving l.s. planes.
Refinement. Refinement of *F*^2^ against ALL reflections. The weighted *R*-factor *wR* and goodness of fit *S* are based on *F*^2^, conventional *R*-factors *R* are based on *F*, with *F* set to zero for negative *F*^2^. The threshold expression of *F*^2^ > σ(*F*^2^) is used only for calculating *R*-factors(gt) *etc*. and is not relevant to the choice of reflections for refinement. *R*-factors based on *F*^2^ are statistically about twice as large as those based on *F*, and *R*- factors based on ALL data will be even larger.

### Fractional atomic coordinates and isotropic or equivalent isotropic displacement parameters (Å^2^)

**Table:**

	*x*	*y*	*z*	*U*_iso_*/*U*_eq_	
Cl1	0.47005 (4)	−0.34472 (13)	0.16802 (5)	0.0679 (3)	
N1	0.04965 (12)	1.3024 (4)	−0.03178 (14)	0.0492 (6)	
N2	0.25468 (11)	0.4873 (4)	0.02923 (14)	0.0459 (5)	
O1	0.01107 (10)	1.4271 (3)	−0.11173 (11)	0.0660 (6)	
H1	−0.0105	1.5364	−0.0895	0.099*	
O2	0.29783 (10)	0.2627 (3)	−0.12216 (12)	0.0714 (6)	
H2	0.2760	0.3628	−0.0934	0.107*	
C1	0.08207 (16)	1.0646 (5)	−0.16879 (17)	0.0649 (9)	
H1A	0.0436	1.1557	−0.2087	0.097*	
H1B	0.0670	0.9107	−0.1768	0.097*	
H1C	0.1344	1.0862	−0.1903	0.097*	
C2	0.08394 (13)	1.1288 (4)	−0.06072 (16)	0.0397 (6)	
C3	0.12822 (12)	0.9899 (4)	0.01858 (16)	0.0368 (6)	
C4	0.16997 (12)	0.8025 (4)	−0.00603 (17)	0.0414 (6)	
H4	0.1693	0.7636	−0.0734	0.050*	
C5	0.21285 (13)	0.6705 (4)	0.06599 (18)	0.0403 (6)	
C6	0.21285 (14)	0.7264 (5)	0.16624 (18)	0.0512 (7)	
H6	0.2406	0.6389	0.2159	0.061*	
C7	0.17157 (15)	0.9121 (5)	0.19178 (18)	0.0552 (8)	
H7	0.1719	0.9499	0.2592	0.066*	
C8	0.12969 (14)	1.0435 (4)	0.11943 (17)	0.0465 (7)	
H8	0.1023	1.1688	0.1384	0.056*	
C9	0.29415 (13)	0.3476 (4)	0.08762 (19)	0.0462 (7)	
H9	0.2948	0.3651	0.1567	0.055*	
C10	0.33755 (13)	0.1644 (4)	0.04994 (18)	0.0410 (6)	
C11	0.33784 (14)	0.1268 (5)	−0.05337 (19)	0.0491 (7)	
C12	0.37878 (14)	−0.0525 (5)	−0.08613 (19)	0.0572 (8)	
H12	0.3789	−0.0767	−0.1547	0.069*	
C13	0.41961 (14)	−0.1967 (5)	−0.0189 (2)	0.0547 (7)	
H13	0.4473	−0.3176	−0.0417	0.066*	
C14	0.41926 (13)	−0.1606 (4)	0.08297 (19)	0.0459 (7)	
C15	0.37940 (13)	0.0169 (4)	0.11697 (18)	0.0462 (7)	
H15	0.3801	0.0399	0.1857	0.055*	

### Atomic displacement parameters (Å^2^)

**Table:**

	*U*^11^	*U*^22^	*U*^33^	*U*^12^	*U*^13^	*U*^23^
Cl1	0.0726 (5)	0.0574 (5)	0.0723 (5)	0.0192 (4)	0.0021 (4)	0.0118 (4)
N1	0.0631 (13)	0.0413 (15)	0.0398 (12)	0.0115 (12)	−0.0090 (11)	0.0044 (11)
N2	0.0427 (12)	0.0397 (15)	0.0543 (13)	0.0036 (11)	0.0008 (10)	0.0030 (11)
O1	0.0957 (14)	0.0518 (14)	0.0464 (11)	0.0267 (11)	−0.0102 (10)	0.0062 (9)
O2	0.0864 (13)	0.0763 (16)	0.0495 (11)	0.0282 (12)	−0.0015 (10)	0.0080 (10)
C1	0.086 (2)	0.070 (2)	0.0373 (15)	0.0267 (17)	0.0000 (15)	0.0016 (14)
C2	0.0442 (14)	0.0391 (17)	0.0347 (14)	0.0009 (13)	−0.0003 (12)	0.0000 (12)
C3	0.0392 (13)	0.0343 (17)	0.0363 (14)	−0.0022 (12)	0.0019 (11)	0.0010 (12)
C4	0.0453 (14)	0.0431 (18)	0.0346 (13)	−0.0041 (13)	−0.0005 (12)	−0.0007 (12)
C5	0.0395 (14)	0.0344 (16)	0.0465 (16)	−0.0016 (12)	0.0023 (12)	0.0040 (13)
C6	0.0591 (16)	0.050 (2)	0.0437 (16)	0.0102 (14)	0.0013 (13)	0.0107 (13)
C7	0.0703 (18)	0.060 (2)	0.0348 (15)	0.0112 (16)	0.0026 (14)	0.0027 (14)
C8	0.0553 (15)	0.0440 (18)	0.0404 (15)	0.0106 (13)	0.0060 (13)	0.0005 (13)
C9	0.0453 (15)	0.0417 (18)	0.0503 (15)	−0.0006 (14)	0.0000 (13)	−0.0003 (13)
C10	0.0378 (13)	0.0352 (16)	0.0495 (16)	−0.0004 (12)	0.0019 (12)	−0.0013 (13)
C11	0.0448 (15)	0.054 (2)	0.0474 (17)	0.0058 (14)	−0.0004 (13)	0.0054 (14)
C12	0.0594 (17)	0.068 (2)	0.0447 (16)	0.0077 (16)	0.0070 (14)	−0.0040 (15)
C13	0.0473 (15)	0.053 (2)	0.0645 (19)	0.0076 (14)	0.0098 (15)	−0.0063 (15)
C14	0.0397 (14)	0.0403 (18)	0.0567 (17)	0.0044 (13)	0.0009 (13)	0.0048 (14)
C15	0.0425 (14)	0.0480 (19)	0.0465 (15)	−0.0007 (13)	−0.0016 (12)	0.0002 (13)

### Geometric parameters (Å, °)

**Table:**

Cl1—C14	1.739 (2)	C5—C6	1.384 (3)
N1—C2	1.269 (3)	C6—C7	1.373 (3)
N1—O1	1.403 (2)	C6—H6	0.9300
N2—C9	1.279 (3)	C7—C8	1.379 (3)
N2—C5	1.419 (3)	C7—H7	0.9300
O1—H1	0.8200	C8—H8	0.9300
O2—C11	1.351 (3)	C9—C10	1.439 (3)
O2—H2	0.8200	C9—H9	0.9300
C1—C2	1.495 (3)	C10—C15	1.393 (3)
C1—H1A	0.9600	C10—C11	1.402 (3)
C1—H1B	0.9600	C11—C12	1.372 (3)
C1—H1C	0.9600	C12—C13	1.375 (3)
C2—C3	1.482 (3)	C12—H12	0.9300
C3—C4	1.382 (3)	C13—C14	1.381 (3)
C3—C8	1.386 (3)	C13—H13	0.9300
C4—C5	1.385 (3)	C14—C15	1.361 (3)
C4—H4	0.9300	C15—H15	0.9300
			
C2—N1—O1	112.91 (19)	C6—C7—H7	119.4
C9—N2—C5	122.4 (2)	C8—C7—H7	119.4
N1—O1—H1	109.5	C7—C8—C3	120.3 (2)
C11—O2—H2	109.5	C7—C8—H8	119.8
C2—C1—H1A	109.5	C3—C8—H8	119.8
C2—C1—H1B	109.5	N2—C9—C10	122.2 (2)
H1A—C1—H1B	109.5	N2—C9—H9	118.9
C2—C1—H1C	109.5	C10—C9—H9	118.9
H1A—C1—H1C	109.5	C15—C10—C11	118.5 (2)
H1B—C1—H1C	109.5	C15—C10—C9	119.8 (2)
N1—C2—C3	116.7 (2)	C11—C10—C9	121.7 (2)
N1—C2—C1	123.0 (2)	O2—C11—C12	118.8 (2)
C3—C2—C1	120.3 (2)	O2—C11—C10	121.4 (2)
C4—C3—C8	117.8 (2)	C12—C11—C10	119.8 (2)
C4—C3—C2	120.8 (2)	C11—C12—C13	120.8 (2)
C8—C3—C2	121.4 (2)	C11—C12—H12	119.6
C3—C4—C5	122.4 (2)	C13—C12—H12	119.6
C3—C4—H4	118.8	C12—C13—C14	119.5 (3)
C5—C4—H4	118.8	C12—C13—H13	120.3
C6—C5—C4	118.8 (2)	C14—C13—H13	120.3
C6—C5—N2	125.2 (2)	C15—C14—C13	120.6 (2)
C4—C5—N2	116.0 (2)	C15—C14—Cl1	120.0 (2)
C7—C6—C5	119.5 (2)	C13—C14—Cl1	119.5 (2)
C7—C6—H6	120.3	C14—C15—C10	120.7 (2)
C5—C6—H6	120.3	C14—C15—H15	119.6
C6—C7—C8	121.3 (2)	C10—C15—H15	119.6
			
O1—N1—C2—C3	178.37 (18)	C2—C3—C8—C7	−179.7 (2)
O1—N1—C2—C1	−0.7 (3)	C5—N2—C9—C10	−178.9 (2)
N1—C2—C3—C4	−177.5 (2)	N2—C9—C10—C15	−179.8 (2)
C1—C2—C3—C4	1.6 (3)	N2—C9—C10—C11	−1.0 (4)
N1—C2—C3—C8	2.1 (3)	C15—C10—C11—O2	179.4 (2)
C1—C2—C3—C8	−178.8 (2)	C9—C10—C11—O2	0.6 (4)
C8—C3—C4—C5	−0.5 (3)	C15—C10—C11—C12	0.1 (4)
C2—C3—C4—C5	179.1 (2)	C9—C10—C11—C12	−178.8 (2)
C3—C4—C5—C6	1.0 (3)	O2—C11—C12—C13	−179.3 (2)
C3—C4—C5—N2	−178.2 (2)	C10—C11—C12—C13	0.1 (4)
C9—N2—C5—C6	3.5 (4)	C11—C12—C13—C14	0.1 (4)
C9—N2—C5—C4	−177.4 (2)	C12—C13—C14—C15	−0.5 (4)
C4—C5—C6—C7	−0.9 (4)	C12—C13—C14—Cl1	179.35 (19)
N2—C5—C6—C7	178.2 (2)	C13—C14—C15—C10	0.6 (4)
C5—C6—C7—C8	0.3 (4)	Cl1—C14—C15—C10	−179.18 (17)
C6—C7—C8—C3	0.2 (4)	C11—C10—C15—C14	−0.4 (4)
C4—C3—C8—C7	−0.1 (3)	C9—C10—C15—C14	178.4 (2)

### Hydrogen-bond geometry (Å, °)

**Table:**

*D*—H···*A*	*D*—H	H···*A*	*D*···*A*	*D*—H···*A*
O2—H2···N2	0.82	1.87	2.601 (3)	147
O1—H1···N1^i^	0.82	2.06	2.789 (3)	149

Symmetry codes: (i) −*x*, −*y*+3, −*z*.
